# Supplementary figures and images for: Basal leakage in oscillation: Coupled transcriptional and translational control using feed-forward loops
Source: PLoS Comput Biol. 2020 Sep 3;16(9):e1007740. doi: 10.1371/journal.pcbi.1007740 (PMC7494099; doi:10.1371/journal.pcbi.1007740)

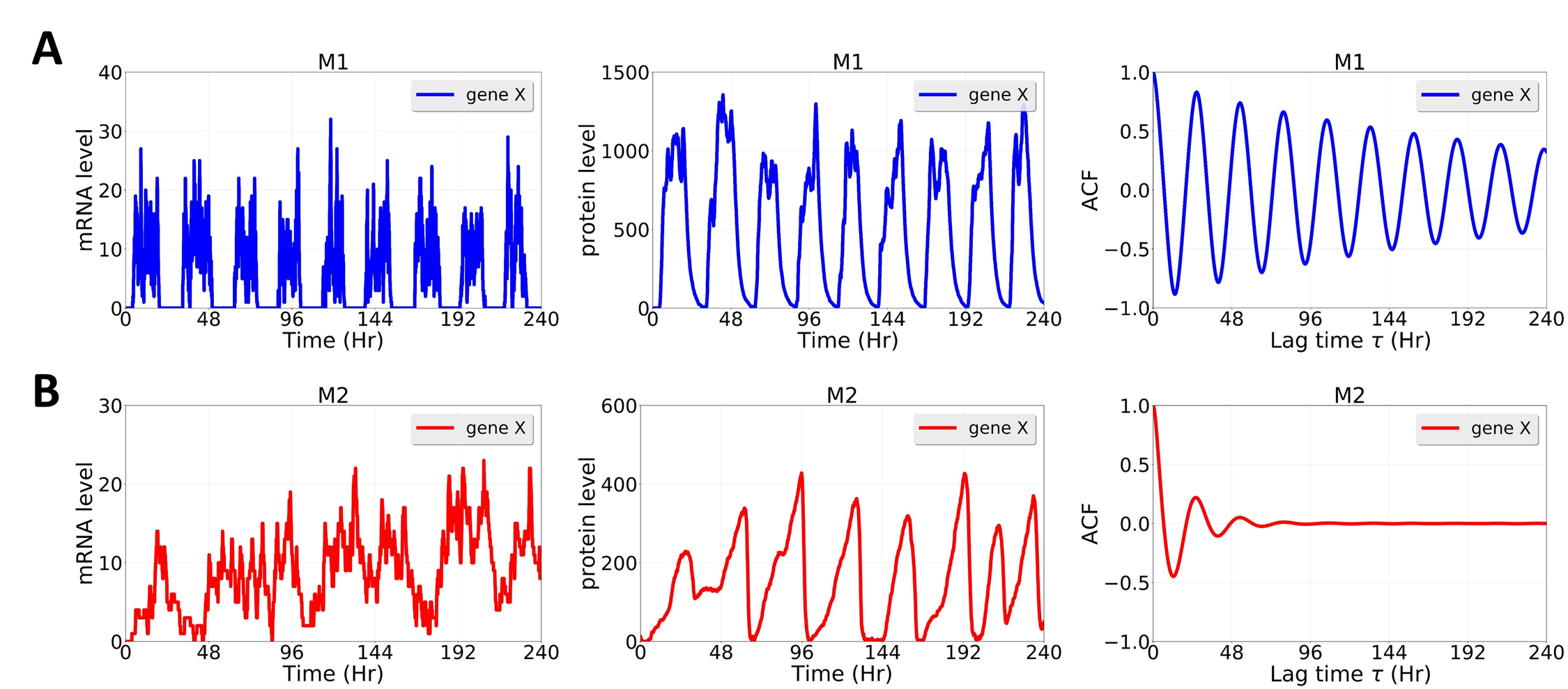

Supplement: S1 Fig — (TIF) [file pcbi.1007740.s001.tif]

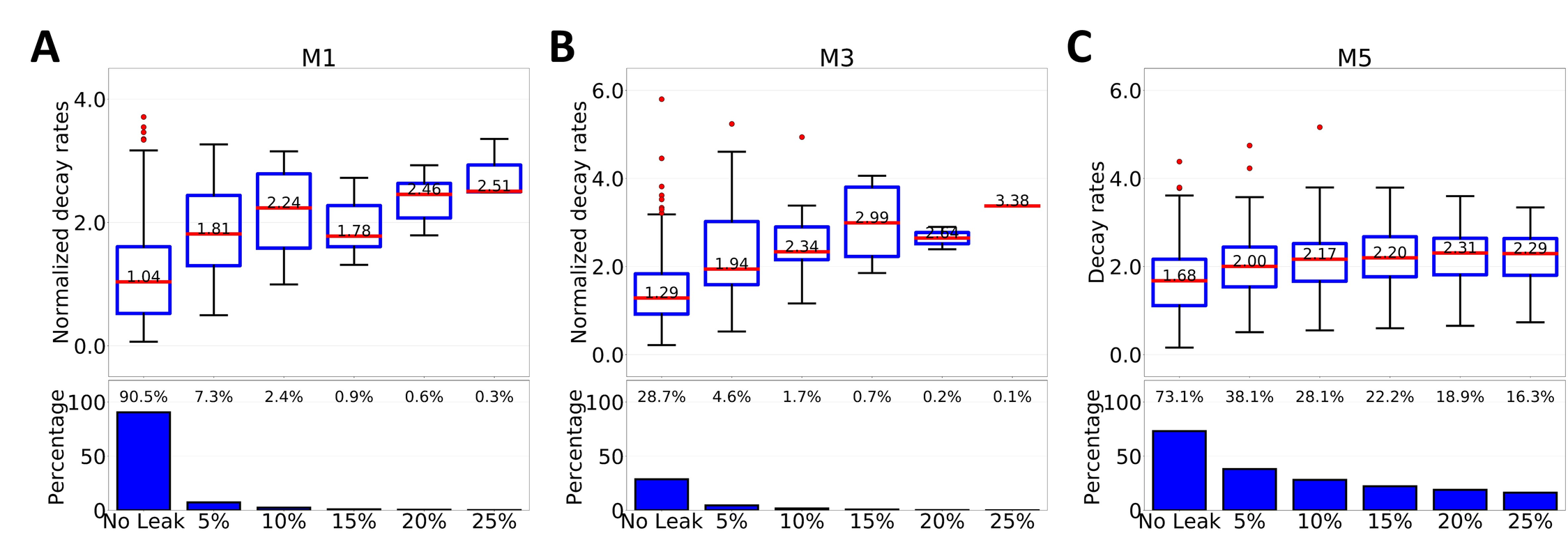

Supplement: S2 Fig — (Upper panels) box plot representing the normalized decay rates for each leakage level of model M1 (A), M3 (B), and M5 (C). (Lower panels) the percentage of parameter sets showing sustained oscillation under stochastic simulation. Red line indicates the median, and box edges indicate the 25th (Q1) and 75th (Q3) percentiles. The whiskers are defined as 1.5*(Q3-Q1). (TIF) [file pcbi.1007740.s002.tif]

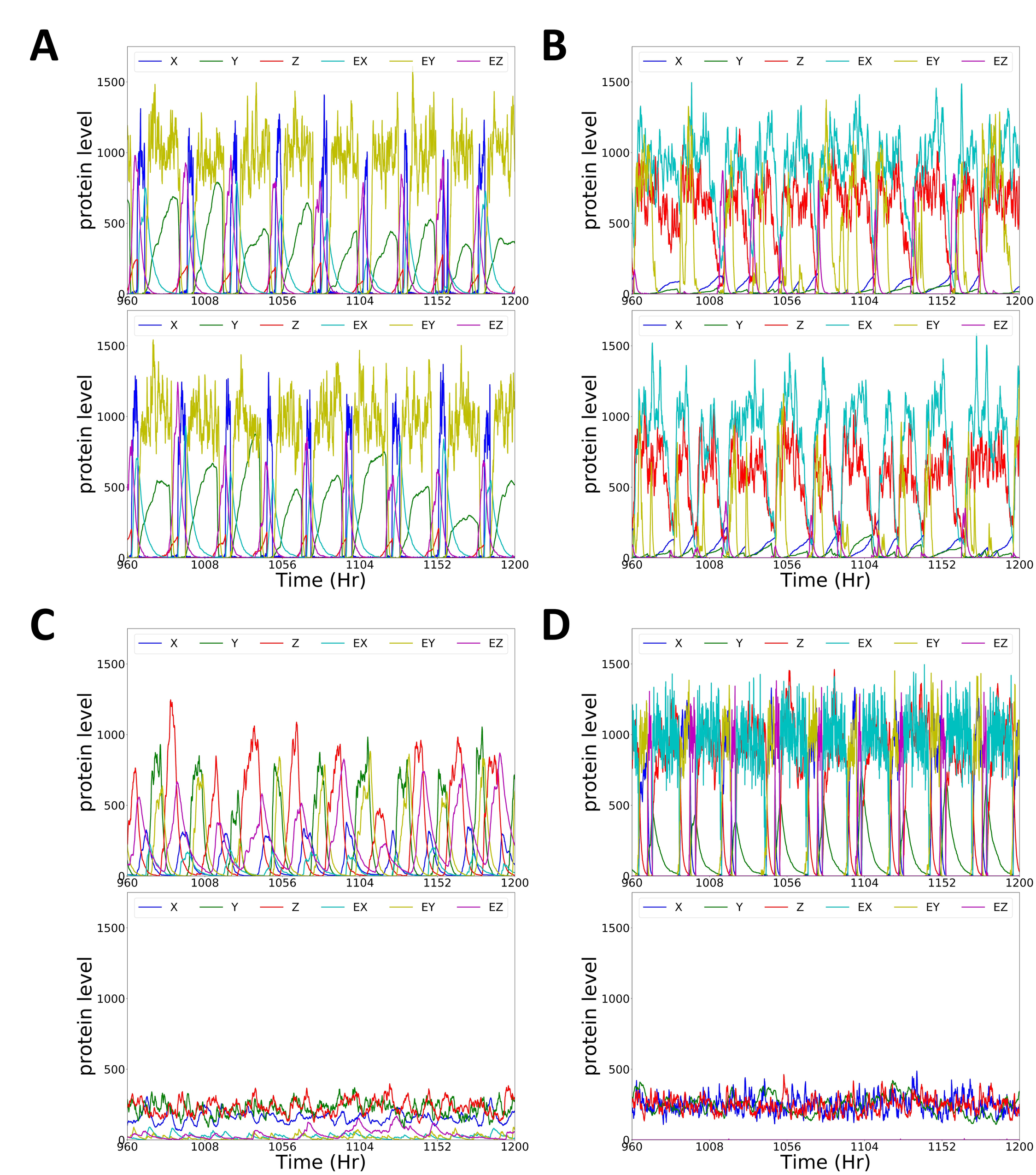

Supplement: S3 Fig — (TIF) [file pcbi.1007740.s003.tif]

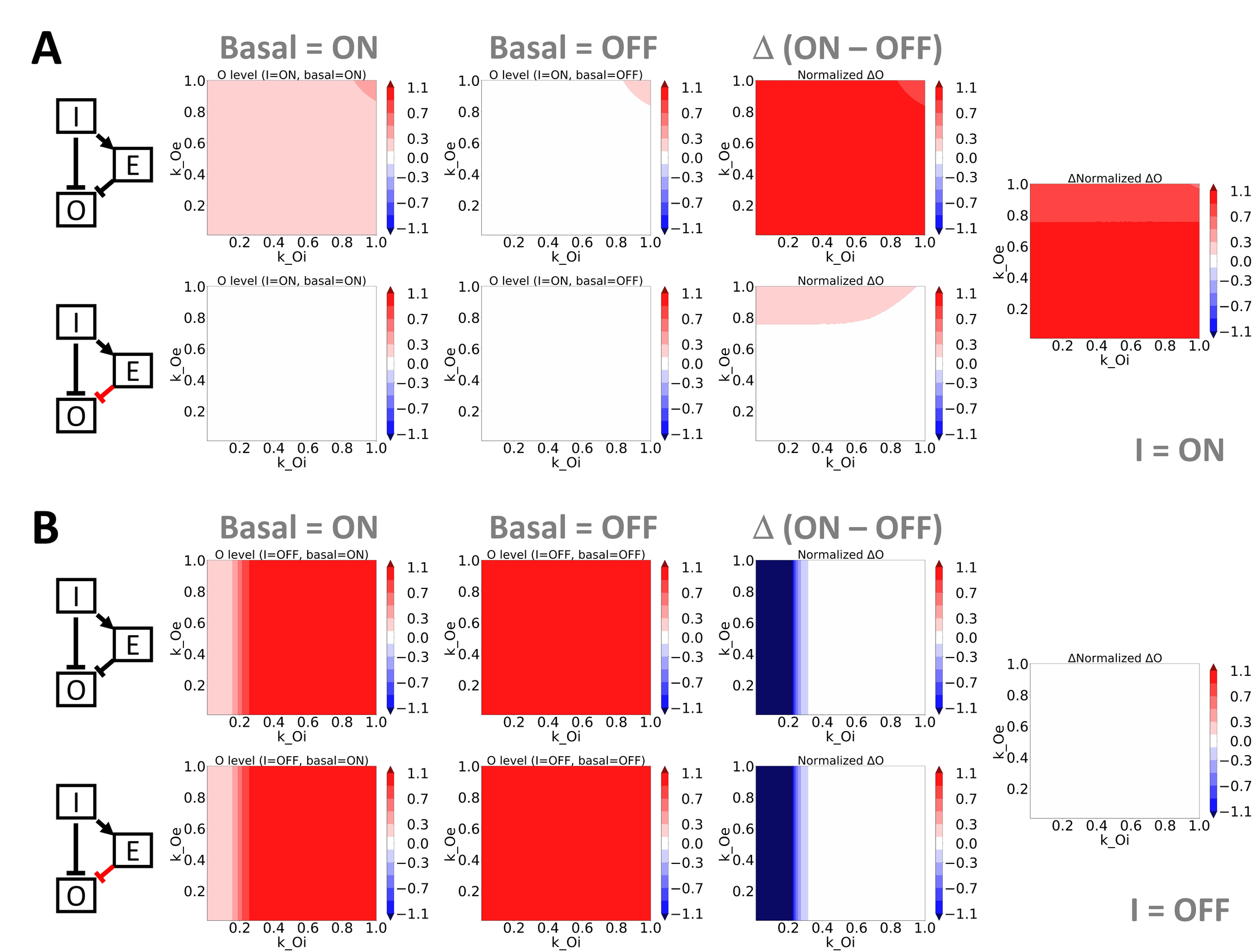

Supplement: S4 Fig — I, E, and O representing the input, intermediate, and output genes, respectively. For simplicity, but without loss of generality, the production and the degradation rates of all genes was fixed at 1, the threshold value of I activation of E (K_Ei) at 0.5, and the Hill coefficient at 8. Furthermore, the leakage level (when Basal = ON) was fixed at 0.2 (or 20% of the maximum possible steady state). (TIF) [file pcbi.1007740.s004.tif]

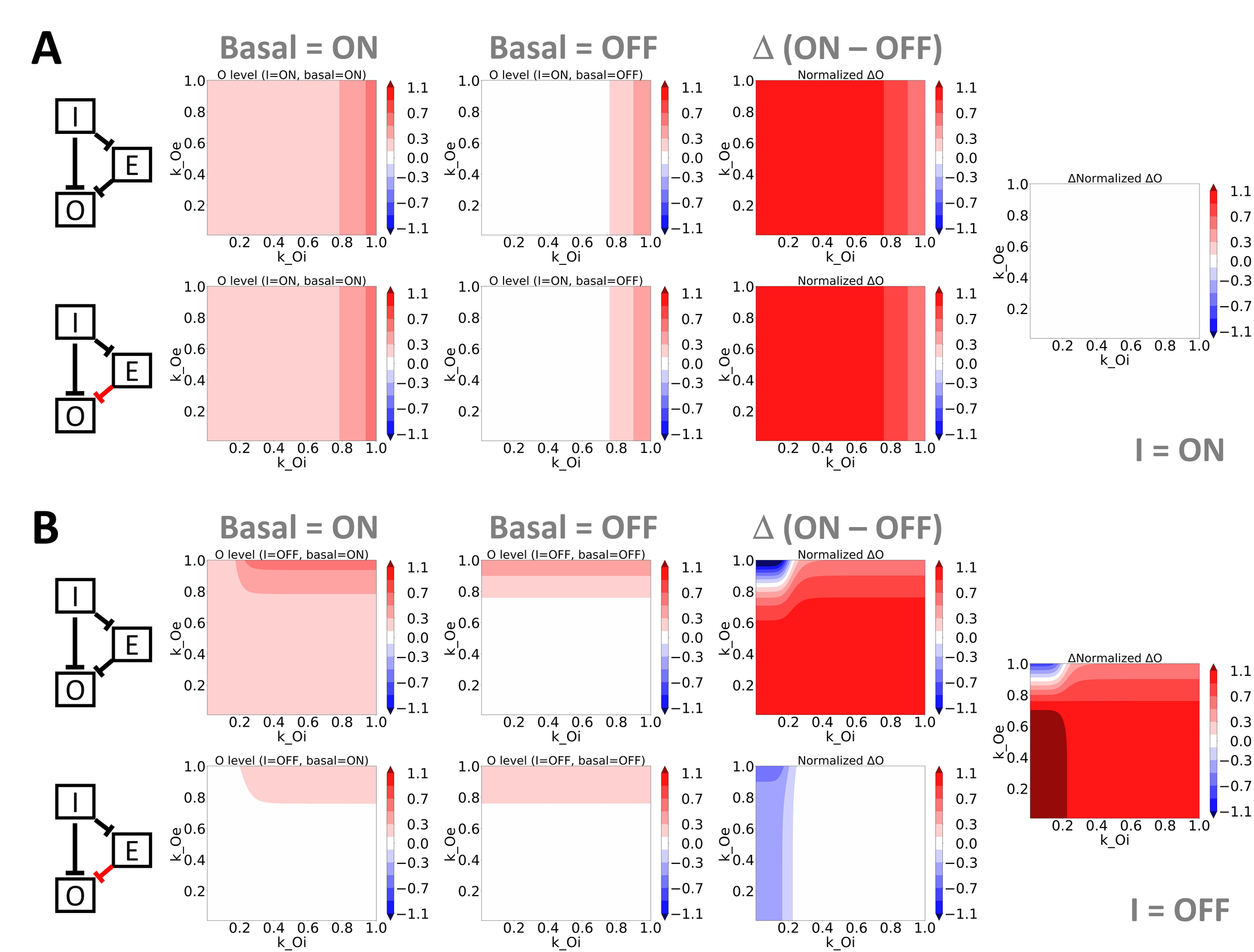

Supplement: S5 Fig — I, E, and O represent the input, intermediate, and output genes, respectively. For simplicity, but without loss of generality, the production and the degradation rates of all genes was fixed at 1, the threshold value of I inhibition of E (K_Ei) at 0.5, and the Hill coefficient at 8. Furthermore, the leakage level (when Basal = ON) was fixed at 0.2 (or 20% of the maximum possible steady state). (TIF) [file pcbi.1007740.s005.tif]

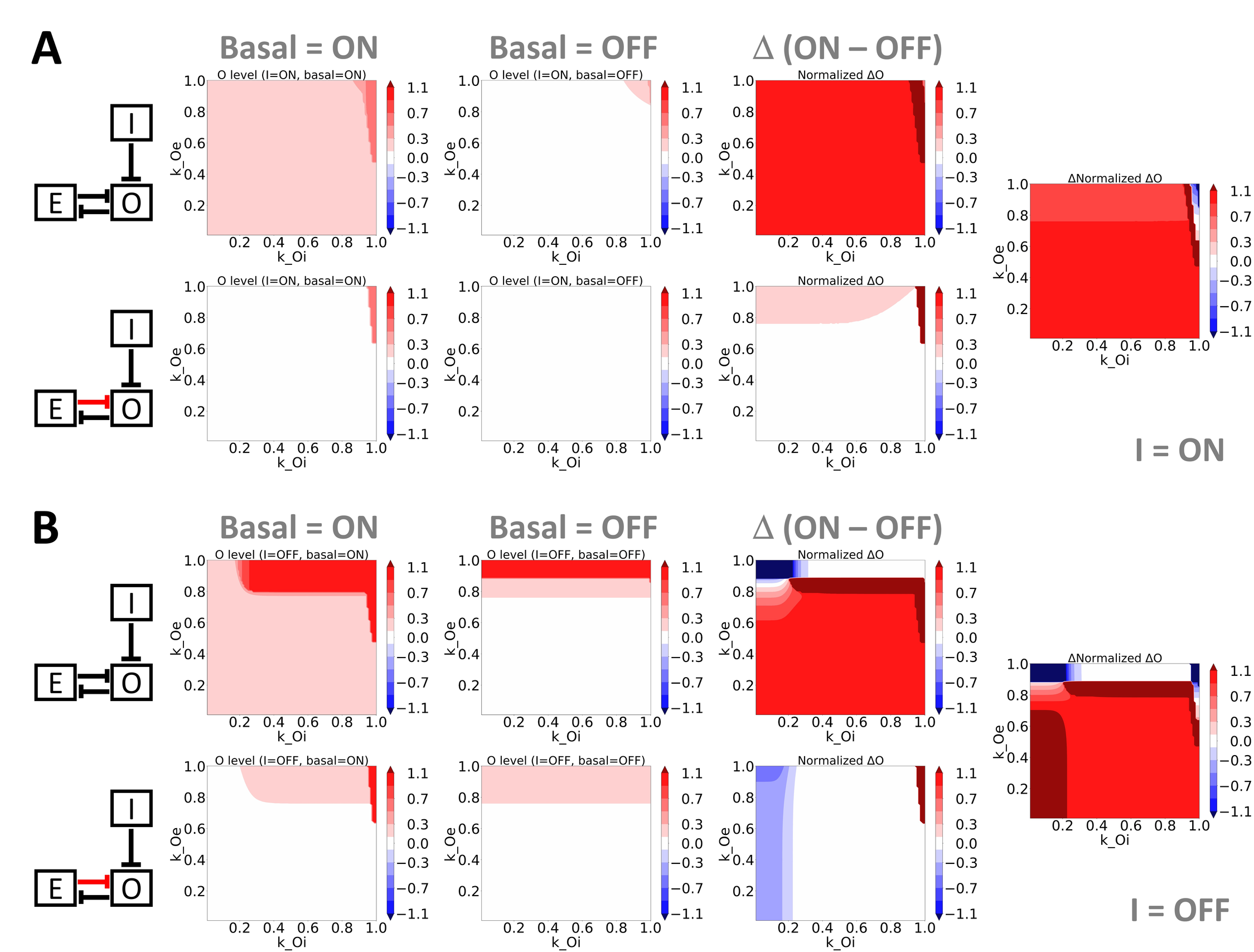

Supplement: S6 Fig — I, E, and O represent the input, intermediate, and output genes, respectively. For simplicity, but without loss of generality, the production and the degradation rates of all genes was fixed at 1, the threshold value of O inhibition of E (K_Eo) at 0.5, and the Hill coefficient at 8. Furthermore, the leakage level (when Basal = ON) was fixed at 0.2 (or 20% of the maximum possible steady state). (TIF) [file pcbi.1007740.s006.tif]

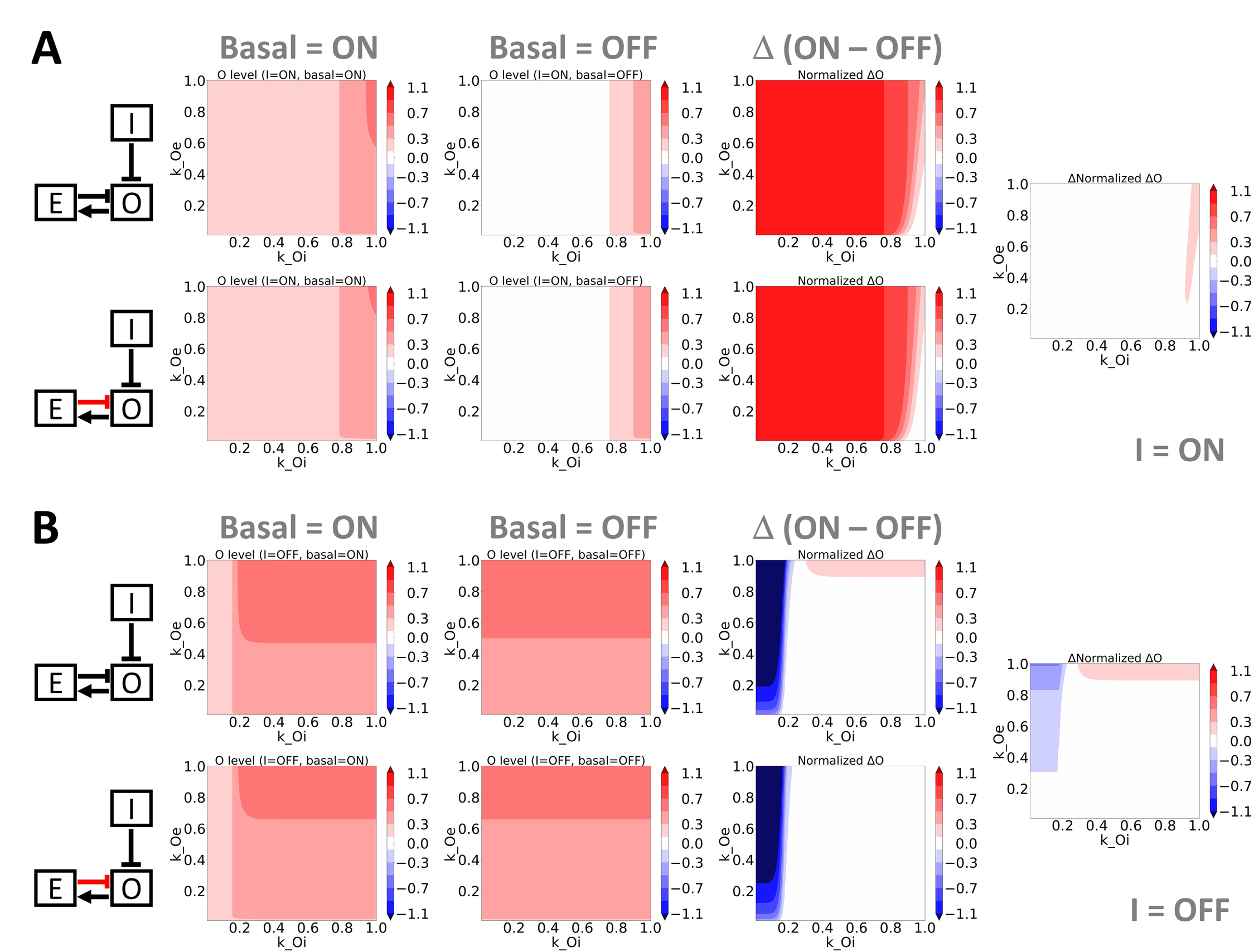

Supplement: S7 Fig — I, E, and O represent the input, intermediate, and output genes, respectively. For simplicity, but without loss of generality, the production and the degradation rates of all genes was fixed at 1, the threshold value of O activation of E (K_Eo) at 0.5, and the Hill coefficient at 8. Furthermore, the leakage level (when Basal = ON) was fixed at 0.2 (or 20% of the maximum possible steady state). (TIF) [file pcbi.1007740.s007.tif]

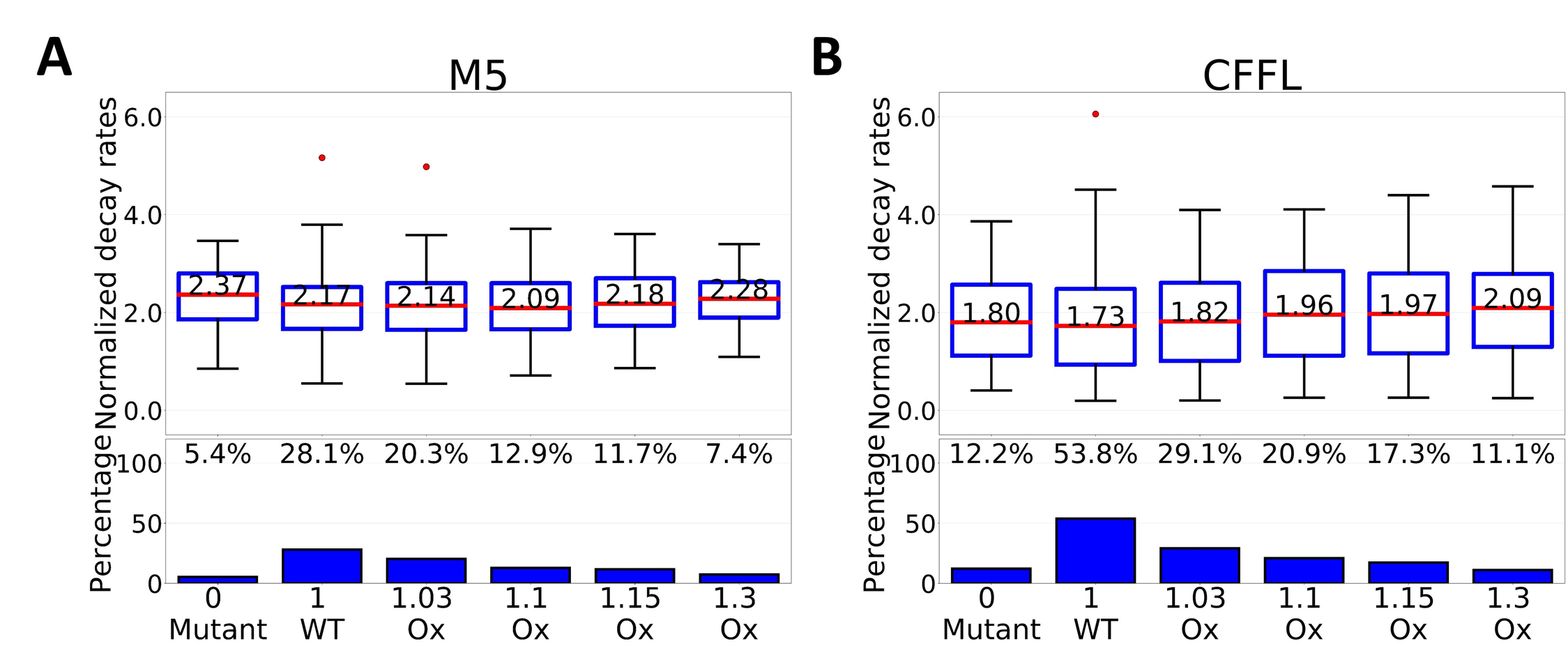

Supplement: S8 Fig — (Upper panel) box plot representing the normalized decay rates for each mutant condition of model M5 (A), and CFFL (B). Red line indicates the median, and box edges indicate the 25th (Q1) and 75th (Q3) percentiles. The whiskers are defined as 1.5*(Q3-Q1). (Lower panel) The percentage of parameter sets showing sustained oscillation under stochastic simulation. (TIF) [file pcbi.1007740.s008.tif]

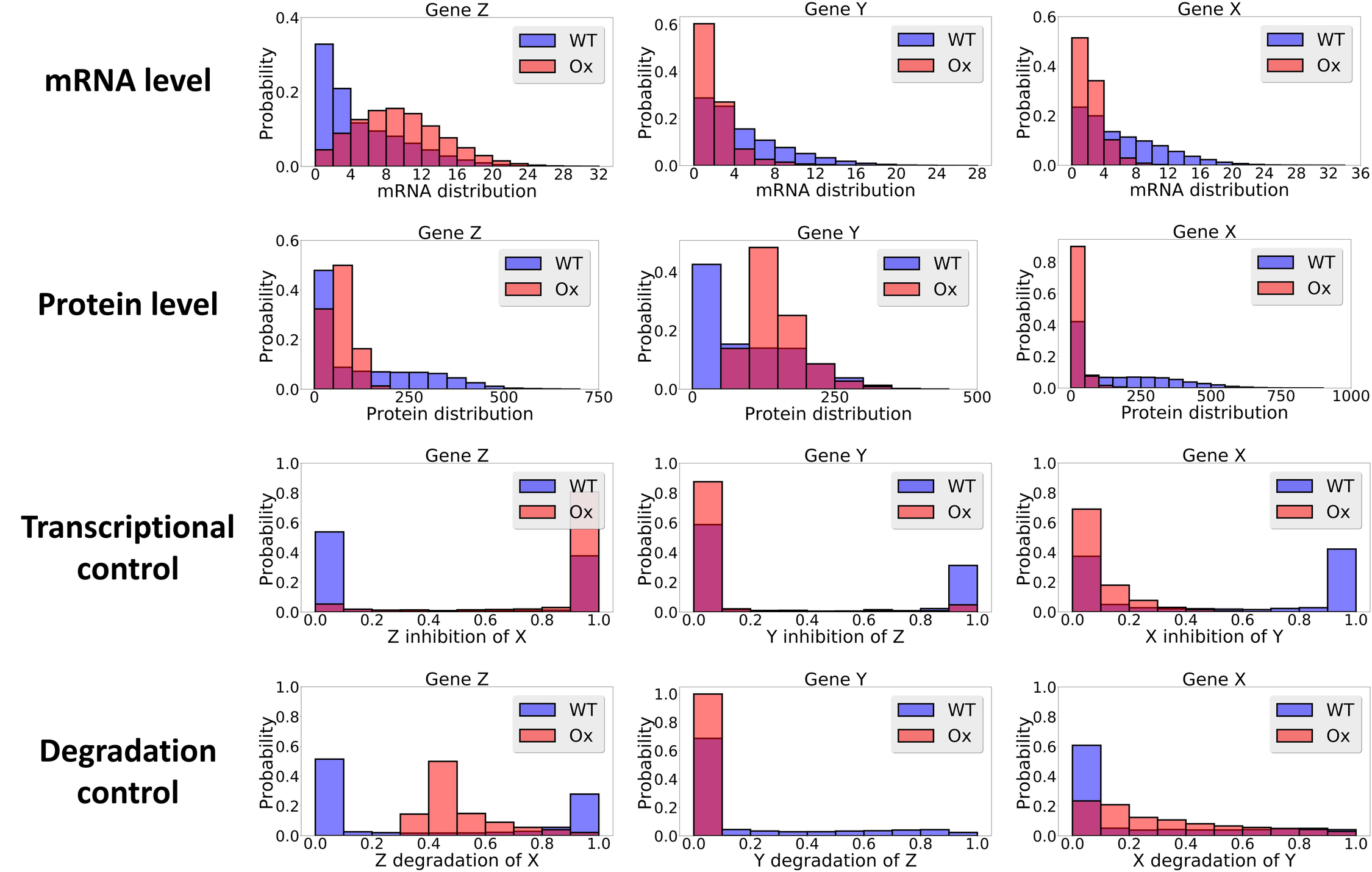

Supplement: S9 Fig — The distribution was collected from the wild type (WT) (blue) or over-expression condition (red) with 10% basal leakage. (TIF) [file pcbi.1007740.s009.tif]
