# Supplementary material for: Basal leakage in oscillation: Coupled transcriptional and translational control using feed-forward loops
Source: PLoS Comput Biol. 2020 Sep 3;16(9):e1007740. doi: 10.1371/journal.pcbi.1007740 (PMC7494099; doi:10.1371/journal.pcbi.1007740)
Supplement: S1 Appendix — (ZIP) [file pcbi.1007740.s010.zip › Codes/Readme.pdf]

For each model, there will be few file:

1. Code for randomly searched the parameter sets ("1_*.py"). The output of this code is a list of parameter sets and a list of oscillation properties in csv format.
2. Code for performing SSA simulation and autocorrelation functions ("2_.py"). The output of this code is an average autocorrelation function for each parameter sets.
3. Code for calculating decay rates ("3_.py"). The output of this code is a list of decay rates for parameter sets that was considered oscillating during SSA simulation.

For model M1 and M5, there are some additional code:

1. Code for calculating distribution of the oscillation for all parameter sets ("4a_.py")
2. Code for calculating KL-divergence for all parameter sets ("4b_.py")

For all model, the stochastic simulation was done using C on python (Cython) to speed up the simulation process. Before it can be used, this "_.pyx" files need to be compiled first. To compile cython file ("_.pyx") in windows, make sure that windows compilers (Microsoft Visual C++ 14.2 standalone: Build Tools for Visual Studio 2019) are installed. Here some information about installing window installer (https://wiki.python.org/moin/WindowsCompilers)

Finally, we also provide all codes and data needed to redraw all figures in the main text (under plots folder).

If you have further question, feel free to contact us at (cherri@sinica.edu.tw or ignasiusjoanito@hotmail.com)
