# Supplementary material for: Basal leakage in oscillation: Coupled transcriptional and translational control using feed-forward loops
Source: PLoS Comput Biol. 2020 Sep 3;16(9):e1007740. doi: 10.1371/journal.pcbi.1007740 (PMC7494099; doi:10.1371/journal.pcbi.1007740)
Supplement: Attachment — Submitted filename: PC_Joanito.pdf [file pcbi.1007740.s011.pdf]

The manuscript titled “Basal leakage in oscillation: coupled transcriptional and translational control using feed-forward loops ” identifies how coupling post-translational control with transcriptional-based oscillator in a feed-forward loop improves the robustness of the oscillator in the presence of basal leakage by building a series of simple models based on the repressilator with different post-translational regulations and comparing their decay rate of an autocorrelation function. Importantly, this explains the increased variability observed in the E3 ligase mutant for both plant and mammalian clocks and the role of existing feed-forward loops in the core feedback loop of the plant clock. Considering the impact and novelty of the findings in this work, I strongly believe that the manuscript fits well with PLOS Computational Biology.

Major comments

1. Fig. 1A: Unlike transcriptional regulation, post-translational regulation is described by both liner and hill-type functions. Do the results of Fig. 1BC depend on the presence of the linear degradation terms?
2. In this study, Hill-type functions are used to describe the transcriptional regulation. However, in the mammalian/Drosophila clock, the transcriptional regulation occurs via protein sequestration (Kim and Forger, Mol Syst Biol 2012 and Kim JK, IET Syst Biol 2016). These studies show that unlike the Hill-type regulation, with transcriptional repression via protein sequestration, the additional negative feedback loop rather than positive feedback loop enhances the robustness of rhythms in contrast to Fig. 3B. As the manuscript considers the mammalian circadian clock as well, it would be great if the authors can discuss whether the finding can be extendable to the system whose transcriptional repression occurs via protein sequestration in the discussion section.
3. L568: In this study, stochastic simulations are performed with the propensity functions of Hill-functions. As the Hill-functions are derived with the deterministic QSSA, stochastic simulations with the Hill-functions can cause serious errors in the fluctuation level of the system (Thomas et al, BMC Syst Biol (2012), Kim, Josic, Bennett, BMC Syst Biol (2015), Kim and Sontag, PLOS Com Biol (2017)). However, it would be computationally prohibitive that stochastic simulations are reperformed after converting the Hill functions to a series of

mass-action kinetics in this work. But, at least the potential limitation of the approach used in this study should be stated for readers.

4. The dosage-dependent effect of ZTL on circadian rhythms are measured via relative amplitude error. However, in the manuscript a decay rate of an autocorrelation function is used for the comparison (Fig. 5A/C). Do the relative amplitude error and the autocorrelation decay rate lead to the same conclusion?

Minor comments

1. L139: Some parameter sets generating oscillation with the deterministic model do not yield the oscillations in the stochastic model. How does this happen? Usually, the stochasticity prevents the damping of oscillations and helps sustain oscillations.
2. Two different notations (CFFL and FFL) are used for the same network, which is confusing.
3. L263: Clear explanation is needed for among which I, E, O, the basal is directly involved. I guess O?
4. Fig. 4D: When the basal is on, the O increases between t=20 and 40. Thus, E should increase as O induces E. But, there is no change in E. How?
5. L328: Will-type=> Wild-type.
6. Revise G to G_x in Eq (39) Furthermore, please explicitly express the meaning of G_x.
7. L265 & Figure 3A legend: 'translation control' or ' post-translational control'?
8. Fig. 3C: Change the position of PF and NF graphs so that the order of graphs is consistent with Fig. 3A/B.
