# Supplementary material for: Basal leakage in oscillation: Coupled transcriptional and translational control using feed-forward loops
Source: PLoS Comput Biol. 2020 Sep 3;16(9):e1007740. doi: 10.1371/journal.pcbi.1007740 (PMC7494099; doi:10.1371/journal.pcbi.1007740)
Supplement: Attachment — Submitted filename: Response to reviewers_final.docx [file pcbi.1007740.s012.docx]

**Reviewer 1:**

The manuscript titled “Basal leakage in oscillation: coupled transcriptional and translational control using feed-forward loops” identifies how coupling posttranslational control with transcriptional-based oscillator in a feed-forward loop improves the robustness of the oscillator in the presence of basal leakage by building a series of simple models based on the repressilator with different post-translational regulations and comparing their decay rate of an autocorrelation function. Importantly, this explains the increased variability observed in the E3 ligase mutant for both plant and mammalian clocks and the role of existing feed-forward loops in the core feedback loop of the plant clock. Considering the impact and novelty of the findings in this work, I strongly believe that the manuscript fits well with PLOS Computational Biology.

Major comments

1. Fig. 1A: Unlike transcriptional regulation, post-translational regulation is described by both liner and hill-type functions. Do the results of Fig. 1BC depend on the presence of the linear degradation terms?

**Author reply:**

Conceptually, results in figure 1BC will still be the same with or without basal degradation term, because the overall degradation is predominantly determined by the hill term (the basal degradation is much slower). However, by adding the basal term we can obtain several nice properties such as: first, the model become general because it can also represent other post-translational models (1), and second, the parameter space become more uniform. With this linear degradation term, we can determine the maximum steady state in advance, which help us choosing reasonable range for other parameters such as Kappas. Furthermore, from biological point of view, this linear term can represent a much slower degradation (like “natural” decay rate of protein) or a dilution process due to cell growth. Hence, we decided to keep this basal degradation in our simulation.

2. In this study, Hill-type functions are used to describe the transcriptional regulation. However, in the mammalian/Drosophila clock, the transcriptional regulation occurs via protein sequestration (Kim and Forger, Mol Syst Biol 2012 and Kim JK, IET Syst Biol 2016). These studies show that unlike the Hill-type regulation, with transcriptional repression via protein sequestration, the additional negative feedback loop rather than positive feedback loop enhances the robustness of rhythms in contrast to Fig. 3B. As the manuscript considers the mammalian circadian clock as well, it would be great if the authors can discuss whether the finding can be extendable to the system whose transcriptional repression occurs via protein sequestration in the discussion section.

**Author reply:**

We are aware of different formulation of transcriptional process, such as protein sequestration (2, 3). We believed that our finding will still stand even if we change our regulation term. However, we agree with the reviewer that we should inform our reader about this other regulation form as well. Hence we have added a paragraph in the discussion section describing protein sequestration in mammalian’s clock (L497-504).

3. L568: In this study, stochastic simulations are performed with the propensity functions of Hill-functions. As the Hill-functions are derived with the deterministic QSSA, stochastic simulations with the Hill-functions can cause serious errors in the fluctuation level of the system (Thomas et al, BMC Syst Biol (2012), Kim, Josic, Bennett, BMC Syst Biol (2015), Kim and Sontag, PLOS Com Biol (2017)). However, it would be computationally prohibitive that stochastic simulations are reperformed after converting the Hill functions to a series of mass-action kinetics in this work. But, at least the potential limitation of the approach used in this study should be stated for readers.

**Author reply:**

We thank the reviewer for bringing this issue to our attention. Indeed the employment of the Hill function is to reduce the computational cost of more elaborate mass-action kinetics, but with a price of not able to describe the noises properly due to the nonlinearity. We added two new paragraphs in our discussion section to make sure that our reader also aware of this limitation (L466-485).

4. The dosage-dependent effect of ZTL on circadian rhythms are measured via relative amplitude error. However, in the manuscript a decay rate of an autocorrelation function is used for the comparison (Fig. 5A/C). Do the relative amplitude error and the autocorrelation decay rate lead to the same conclusion?

**Author reply:**

Yes, we believe that using decay rate will lead to the same conclusion as if we were using RAE. Previously, we have performed some tests and found that there is a good correlation between results obtained from decay rates and FFT-NLLS (**BRASS**, <http://millar.bio.ed.ac.uk/PEBrown/BRASS/BrassPage.htm>) (Figure R1). However, we did not use RAE, because as stated in BRASS manual, “FFT-NLLS is specifically designed to process data sets that are relatively short and/or noisy and is generally capable of extracting relatively weak rhythms”, which is commonly seen in experimental data. Since our simulation data can have a very long trajectory (24000 Hr) we believed autocorrelation will give better approximation on the goodness of the oscillation. Hence, we decided to use autocorrelation decay rate instead.

5.

Figure R1. Autocorrelation decay rate and RAE results are correlated. Here, we randomly choose one parameter sets from model M1 and calculate 500 and 10 different trajectories for decay rate and RAE, respectively. For RAE calculation, we only use 240 hr trajectories (t=960-1200), while for autocorrelation decay rates we use 24000 hr trajectories. We cannot calculate to many or to long trajectories for RAE due to BRASS software limitation. Finally, the decay rate term is not normalized with their periods in this analysis.

Minor comments

1. L139: Some parameter sets generating oscillation with the deterministic model do not yield the oscillations in the stochastic model. How does this happen? Usually, the stochasticity prevents the damping of oscillations and helps sustain oscillations.

**Author reply:**

Indeed, stochasticity can prevent the damping of oscillations or even create sustained oscillations in some systems. What we are aware of are systems with “stochastic resonance” which were found in excitable systems like neurons or the P53-Mdm2 oscillation which is in respond to external stimulus and the oscillation does not need to be robust and stable (4-8). This part is related to question 4 of Reviewer 2, and so our reply is similar. In general, we believe that circadian clock’s oscillations should be robust and it should not depend on noise. Hence, picking parameter sets that oscillate during ODE simulations is a reasonable strategy.

Regarding some oscillation in deterministic simulation becoming non-oscillating, since all parameters were randomly selected in this study, it is possible that some oscillations are very sensitive to perturbation. To illustrate it, we picked 1 parameter sets from model M1 that was marked as not oscillating (Figure R2). In this case, the presence of noise prolongs the oscillation periods from 24 hr to more than 480 hr, due to the noise in gene Y (gene Y is slowly degraded, Figure R2 middle). Hence it affected the autocorrelation function significantly as shown in figure R2 right.

Figure R2. Dynamics of one parameter set in M1 that does not oscillate under stochastic simulation. (Left) ODE, (Middle) SSA, and (Right) average of autocorrelation function from 500 different trajectories.

2. Two different notations (CFFL and FFL) are used for the same network, which is confusing.

**Author reply:**

We thank the reviewer for bringing up this problem. We have changed all FFL to CFFL in the manuscript.

3. L263: Clear explanation is needed for among which I, E, O, the basal is directly involved. I guess O?

**Author reply:**

Basal was added in both input (I) and output (O) genes with a goal to test the E (degradation control). We have revised the manuscript and added a sentence explicitly mentioning this setting in the results section (L254-256)

4. Fig. 4D: When the basal is on, the O increases between t=20 and 40. Thus, E should increase as O induces E. But, there is no change in E. How?

**Author reply:**

It was indeed increased but it is not sufficiently visible in the plot. In this setting, we fixed threshold value of O activation of E (K_Eo) at 0.5 and focused our analysis for different threshold value of I inhibition of O (K_Oi) and E inhibition of O (K_Oe) (Figure S4-S7). Because of this reason, the additional basal expression in O (which is only 20%) is not sufficient to activate the expression of E in NF model.

We noticed that we did not mention this setting clearly in the manuscript. Hence, we added few sentences describing this simplification (L256-260)

5. L328: Will-type=> Wild-type.

**Author reply:**

It is revised accordingly.

6. Revise 𝐺 to 𝐺_x_ in Eq (39) Furthermore, please explicitly express the meaning of 𝐺_x_.

**Author reply:**

Previously, we use gene X from model M5 as an example for Eq(39). However, we noticed that it can cause confusion for our reader. Hence, we modified it by changing X into a more general term gene A (which can be X, Y, or Z). the explicit meaning of G_A_ has also been added (L655-661)

7. L265 & Figure 3A legend: ‘translation control’ or ’ post-translational control’?

**Author reply:**

It is the later, ‘post-translation control’, and the manuscript has been revised accordingly

8. Fig. 3C: Change the position of PF and NF graphs so that the order of graphs is consistent with Fig. 3A/B.

**Author reply:**

The manuscript has been revised accordingly

Reviewer 2

The submitted manuscript explores the idea that post-translational controls can soften the negative impact of transcriptional leakage on oscillators. Transcriptional leakage is very common in promoters across different organisms, though it is known that basal production rates reduce the robustness of oscillators to parameter and molecular fluctuations. So when an oscillator is built upon transcriptional control, it is very reasonable to ask whether there is a mechanism in place to compensate for transcriptional leakage. The authors have identified two examples of this in the literature, one in the Arabidopsis circadian clock, and another in the mammalian clock.

I found the overall story to be interesting and a clear hypothesis has been tested.

1. The authors state that the importance of post-translational mechanisms are rarely discussed in studies modelling the Arabidopsis clock. It would be better to substitute this subjective statement for a summary of the instances in which post-translational mechanisms *are* addressed in modelling work. For example, in Pokhilko et al., MSB 2012, the authors incorporate post-translational regulation of ELF3 protein by the E3 ligase COP1. This example is also highly relevant for the submitted manuscript.

**Author reply:**

We have revised our claim that post-translational control has been neglected in previous models of the Arabidopsis clock (L26-29). Now, we emphasize more on the fact that the importance of having combined regulations (transcription and post-translation) is still rarely discussed in clock system.

2. Line 96-104. The observation that mutation of ZTL protein leads to a loss of oscillation period and amplitude robustness is interesting and deserves attention from modelling studies. It would be helpful to review existing theoretical analyses of the influence of degradation on oscillator robustness, beyond circadian oscillators, to help position the results of the submitted manuscript. Examples include:
   a. Wong et al., MSB 2007: <https://doi.org/10.1038/msb4100172>
   b. Krishna et al., PNAS 2006: <https://doi.org/10.1073/pnas.0604085103>
   c. Caicedo-Casso et al., Sci Rep 2015: https://doi.org/10.1038/srep13161

After all, the results of Figures 1-4 are on abstract repressilator networks, as opposed to realistic models of circadian clocks.

**Author reply:**

We have added in our discussion a paragraph discussing several theoretically studies that highlights the importance of degradation control in oscillation system (L441-452)

3. Line 133. The authors should include a bit more detail about the strategy for randomly generating parameter sets. What distributions are used for sampling parameter values, are how are those distributions chosen to be biologically meaningful? I can see from Table 1 that different ranges and scales are used, so I assume the sampling is done assuming uniform and log-uniform distributions. If so, add these details to the Methods section, and reference it accordingly in the Results.

**Author reply:**

We have included reviewer suggestion in our method (L638-640) and results sections (L134)

4. In the first results section, the authors present results on what fraction of deterministic oscillators maintain their rhythmicity in the stochastic setting. I agree that this is interesting from the point of view of understanding how transcriptional noise might adversely affect the performance of an oscillator as a function of the type of inter-component control (transcriptional, post-translational, etc.). However, it does not incorporate any acknowledgement of the potential for noise to enable oscillations for parameter sets which do not oscillate deterministically, which is a well-studied phenomenon in ring oscillators. There is a nice paper on this from Strelkowa & Barahona (ICNAAM 2012, https://doi.org/10.1063/1.4756221), which additionally considers oscillation quality under different system sizes. In light of this, I question whether it is relevant to ask what fraction of deterministic oscillators also oscillate stochastically. In subsequent results, how should one separate the effects of, for example, promoter leakage on deterministic oscillations and stochastic oscillations? Since stochastic oscillations is the setting of interest here, a better comparison of different architectures would be to simply compare what fraction of parameter sets oscillate stochastically, irrespective of deterministic oscillation.

**Author reply:**

Indeed, stochasticity can prevent the damping of oscillations or even create a sustained oscillations in some systems. The Strelkowa & Barahona work mentioned are interesting, and it seems similar to the “stochastic resonance” reported in the literature (4, 5). In biological systems, stochastic resonance was reported mainly in excitable systems like in neurons or the P53-Mdm2 oscillation which is in respond to external stimulus and the oscillation does not need to be robust and stable (6-8). In general, we believe that circadian clock’s oscillations should be robust and it should not depend on noise. Hence, picking parameter sets that oscillate during ODE simulations is a reasonable strategy. Another (minor) reason is that starting from ODE oscillation is computationally much more feasible in terms of massively screening for parameters, and thus it allows a much better coverage in the parameter screening.

5. Line 170. What is meant by “noise level” here? Figure 1D shows that the leakage level increases the normalized decay rate of the autocorrelation function. I don’t see why this is surprising. In the limit as promoters become inceasingly leaky, they become increasingly independent of their regulators, which can only result in unique stable equilibria, and no oscillations.

**Author reply:**

We agree with the reviewer that it is expected to see the oscillations deteriorate once we added basal leakage. However, what was surprising for us is the fact that the system was very sensitive such that adding 5% leakage reduced the number of oscillating parameters sets from 90.5% to 7.3%.

However, we agree with the reviewer that it may confusing for some reader and also the “noise level” is not clear in our previous sentence. Hence, we revised this sentence to be more clear and precise (L170-172).

6. Line 206. The result presented in Figure 2 is interesting, but I wonder how robust it is to parameter uncertainty? In contrast to Figure 1, the results presented only show a single parameter set. By using distance measures for distributions (e.g. KL divergence), one could summarise the effect of leak itself as a distribution over different parameter sets, which would enable a more general statement to be made about the differences between M1 and M5.

**Author reply:**

We thank the reviewer for the nice suggestion. We have calculated the KL divergence for all the parameter sets and discussed it in our manuscript (L207-211 and figure 2C).

7. Line 263. What does “analyzing it locally” mean in this context? I think this can be introduced more clearly. Furthermore, in this paragraph, it would be nice to point out that the parameter dependency of the conclusions has been analysed in the supplementary figures. This is easy to miss. For example, the text preceding the reference to Figure S3 doesn’t mention parameter variation.

**Author reply:**

We have revised our manuscript and removed the “analysing it locally” entirely. We agreed with the reviewer that it may confuse our reader. Furthermore, we have also added an additional sentence describing that the parameter dependency has been analysed (L253-260 and L283-284)

8. Line 285. This section title is not clear. What observed behaviour?

**Author reply:**

We have revised the section title accordingly (L300-301)

9. Line 294. What is the prevailing hypothesis in the literature for explaining how ZTL dosage influences oscillator robustness? The Somers paper doesn’t appear to mention transcriptional leakage, but maybe other studies have tried to explain this. It would be helpful to give greater coverage of the literature surrounding ZTL expression dosage.

**Author reply:**

To the best of our knowledge, there are no specific paper discussing the effect of ZTL level to the observed noisy behaviour of the clock. During this revision period, we have put additional effort to check papers that cited Somers *et al.*’s paper. According to google scholar, Somers *et al.* has been cited 213 times, but most of the paper citing this work talks about the effect of ZTL mutant (longer period), the phenotype of ZTL mutant plants, the detail mechanism of ZTL as proteasome degradation, and the role of ZTL as photoreceptor or gating temperature compensation in the clock systems. So far, the closest work that have similar finding like ours is from D’Alessandro *et al.* 2017 and Clamons and Murray 2018, as we discussed in the manuscript.

10. Line 565: How the simulations were performed needs more detail. Which packages were used for ODE simulation? Or was custom code used? Which algorithms were used? Ideally, the code used to produce the results should be deposited in a public repository, for reproducibility. Perhaps the editor can comment on the PLoS CB policy on reproducibility and code depositing.

**Author reply:**

We have added in supplementary data all the codes we developed in this study. We hope this allow readers to simulate models and evaluate it themselves.

*Additional minor observations*

1. Author summary. 26s -> 26S.

The manuscript has been revised accordingly.

2. Line 97: ZEITLUPE is spelled incorrectly.

The manuscript has been revised accordingly.

3. Line 208: “when the mRNA transcribed is increased” -> “when the rate of mRNA transcription is increased”

The manuscript has been revised accordingly.

4. Line 608: the reference to (96) doesn’t explain the decay of the autocorrelation function. That paper references a previous paper, which does explain it: d'Eysmond et al., Physical Biology 2013 (https://doi.org/10.1088/1478-3975/10/5/056005). Perhaps the d’Eysmond paper is a better citation to use.

We have added the suggested reference in the manuscript

Reviewer 3

In this interesting paper, Joanito et al. explore in simple models the effect of transcriptional leakage on the robustness of oscillations. They provide computational evidence that post-translational control through targeted degradation can help buffer oscillators from the deleterious effects of transcriptional leakage. Although the results are interesting and are of general interest, there are a number of points that need addressing, mainly involving toning down some of the claims.

Major comments:

1. Novelty of results. The authors make a series of models based on the repressilator to generate their results. At a few points the authors suggest that post-translational control has been neglected in previous models of the Arabidopsis clock. The effects of post-translational control involving ZTL and COP1 have been included in previous Arabidopsis models (e.g PMID: 22395476, PMID:25033214). These models examine transcriptional leakage, but it is important to discuss previous examinations of post-translational control accurately, as I am not sure it is correct to say that these connections have been neglected. Additionally, previous work by this study’s authors in the plant circadian clock revealed that transcriptional feedforward loops can increase robustness (PMID:30224713) and in other clock systems feedforward loops have also been modelled (PMID:28007935). It is important that the authors discuss in greater detail how their new work relates to their previous work that found transcriptional feedforward loops improve robustness. They show that post-translational feedback loops are better than transcriptional ones in their model for robustness against transcriptional leakage, but it would be great to have further discussion of how post translational feed forward loops differ from transcriptional feedback loops.

**Author reply:**

We have revised the relevant parts of manuscript regarding the novelty and claims of this work. (L26-29). Now, we emphasize more on the fact that the consequence of having combined regulations (transcription and post-translation) is still rarely discussed.

Next, we have also discussed how post-translational feed forward loop differ from transcriptional feedback loop in our new discussion section (L505-517) as suggested. We hope this section can help our readers to understand the difference between these two works. Furthermore, we also discussed the importance of studying the effect of combining these two regulation in the future study. Currently, it is very unfortunate that there is still a lack of experimental evidence supporting these two prediction. Hence, we cannot make another detailed model without sufficient data on the gene regulation or expression.

2. Over statement of predictions from model. The authors claim that the greater variability observed in the ZTL mutant can be explained by the reduction in the level of posttranslational control. I am confused by these results. These data are from bulk averaging from many plants. Data at the single cell suggests that there is a great deal of variability between single cell clock rhythms (PMID: PMID:29697372). If the ZTL mutation really increased variability at the single cell level (which is presumably what their model is simulating), I would have thought that the rhythms would be damped out at the whole plant, or multiple plant level. The fact that plant single cell rhythms are variable, and that they are comparing a ‘single cell’ model of the plant to bulk averaged data should be discussed, especially given the work is examining noisy gene expression. In general, the predictions from the authors repressilator model should not be over empathised or linked to data too strongly. There are also newer papers examining the effects of ZTL on the plant circadian clock, beyond the Somers et al., 2004 paper. Do they show similar results?

**Author reply:**

Indeed, the gap between the observed ZTL mutant behaviour and our simulated models is pretty large. First of all we were testing very simple models that could have biological implication, as they were not developed to mimic any for a cell or an organism. We described the general behaviour of such simple models, and tried to see if such results can offer insights to understand the role of E3 ligase that promote protein degradation. Therefore the comparison is only phenomenological which has many missing links. Much is unknown between our simple models and the clock dynamics at the single cell or at the bulk level, which prohibit us from doing more realistic and detailed modelling. The description in the connection of our model and the ZTL experiments are now modified (313-316).

In this study, the autocorrelation was calculated for each parameter set, which is derived from averaging many stochastic trajectories, and thus we don’t think it can resemble “single cell” results. By doing so, we obtained an averaged decay rates values, which was then compared with RAE bulk data. What we did here is quite similar with the “pseudobulk” analysis that is commonly done in single cell RNA-seq field (9-11).

To the best of our knowledge, there are no specific paper discussing the effect of ZTL level to the observed noisy behaviour of the clock. During this revision period, we have put additional effort to check papers that cited Somers *et al.*’s paper. According to google scholar, Somers *et al.* has been cited 213 times, but most of the paper citing this work talks about the effect of ZTL mutant (longer period), the phenotype of ZTL mutant plants, the detail mechanism of ZTL as proteasome degradation, and the role of ZTL as photoreceptor or gating temperature compensation in the clock systems. So far, the closest work that have similar finding like ours is from D’Alessandro *et al.* 2017 and Clamons and Murray 2018, as we discussed in the manuscript.

3. Presentation: It would be excellent if more of the time traces of the simulated oscillations were presented in the main figures – to see more of the dynamics and to understand what the post-translational control is doing to the dynamics. Are their general effects that can be understood? Additionally, more examples could be shown in supplemental as well. It would help the reader understand the effects of post-translational control to observe more simulations directly.

**Author reply:**

We agree with reviewer that some of our readers may prefer to see the time trace trajectory of our models. Hence, we have added time trace trajectory of model M1 and M5 in our main text, as well as time trace trajectory of CFFL, IFFL, PF and NF in the supplementary material (Figure S3). In addition to this, we also provide in our supplementary information a code to generate this time trace trajectory for other parameter sets. We hope this adjustment can help our reader understand our work better.

4. Availability of models. Model code and data should be provided to allow readers to simulate models and evaluate themselves.

**Author reply:**

We have added in supplementary data all the code that we used in this study. We hope this can allow readers to simulate models and evaluate it themselves

**References**

1.	Tsai TY, Choi YS, Ma W, Pomerening JR, Tang C, Ferrell JE, Jr. Robust, tunable biological oscillations from interlinked positive and negative feedback loops. Science. 2008;321(5885):126-9.

2.	Kim JK, Forger DB. A mechanism for robust circadian timekeeping via stoichiometric balance. Molecular Systems Biology. 2012;8(1).

3.	D’Alessandro M, Beesley S, Kim JK, Jones Z, Chen R, Wi J, et al. Stability of Wake-Sleep Cycles Requires Robust Degradation of the PERIOD Protein. Current Biology. 2017;27(22):3454-67.e8.

4.	Hanggi P. Stochastic resonance in biology. How noise can enhance detection of weak signals and help improve biological information processing. Chemphyschem : a European journal of chemical physics and physical chemistry. 2002;3(3):285-90.

5.	McDonnell MD, Abbott D. What Is Stochastic Resonance? Definitions, Misconceptions, Debates, and Its Relevance to Biology. PLOS Computational Biology. 2009;5(5):e1000348.

6.	Longtin A. Stochastic resonance in neuron models. Journal of Statistical Physics. 1993;70(1):309-27.

7.	Leenders G, Tuszynski J. Stochastic and Deterministic Models of Cellular p53 Regulation. Frontiers in Oncology. 2013;3(64).

8.	Arora A, Gera S, Maheshwari T, Raghav D, Alam MJ, Singh RKB, et al. The Dynamics of Stress p53-Mdm2 Network Regulated by p300 and HDAC1. PLOS ONE. 2013;8(2):e52736.

9.	Zhang L, Yu X, Zheng L, Zhang Y, Li Y, Fang Q, et al. Lineage tracking reveals dynamic relationships of T cells in colorectal cancer. Nature. 2018;564(7735):268-72.

10.	Madissoon E, Wilbrey-Clark A, Miragaia RJ, Saeb-Parsy K, Mahbubani KT, Georgakopoulos N, et al. scRNA-seq assessment of the human lung, spleen, and esophagus tissue stability after cold preservation. Genome biology. 2019;21(1):1.

11.	Shulse CN, Cole BJ, Ciobanu D, Lin J, Yoshinaga Y, Gouran M, et al. High-Throughput Single-Cell Transcriptome Profiling of Plant Cell Types. Cell reports. 2019;27(7):2241-7.e4.
